# eRF3b, a Biomarker for Hepatocellular Carcinoma, Influences Cell Cycle and Phosphoralation Status of 4E-BP1

**DOI:** 10.1371/journal.pone.0086371

**Published:** 2014-01-23

**Authors:** Man Li, Jian Wang, Lei Yang, Ping Gao, Qing-bao Tian, Dian-wu Liu

**Affiliations:** 1 Department of Epidemiology and Statistic, Hebei Medical University, Shijiazhuang, Hebei Province, China; 2 Department of Epidemiology, Hebei North University, Zhangjiakou, Hebei Province, China; Virginia Commonwealth University, United States of America

## Abstract

**Background:**

Hepatitis B virus (HBV) infection and its sequelae are now recognized as serious problems globally. Our aime is to screen hepatocellular carcinoma (HCC) from chronic hepatitis B (CHB) and identify the characteristics of proteins involved.

**Methodology/Principal Findings:**

We affinity-purified sample serum with weak cation-exchange (WCX) magnetic beads and matrix-assisted laser desorption/ionization time of flight mass spectrometry (MALDI-TOF MS) analysis to search for potential markers. The 4210 Da protein, which differed substantially between HCC and CHB isolates, was later identified to be eukaryotic peptide chain release factor GTP-binding subunit eRF3b. Further research showed that eRF3b/GSPT2 was positively expressed in liver tissues. GSPT2 mRNA was, however differentially expressed in blood. Compared with normal controls, the relative expression of GSPT2/18s rRNA was higher in CHB patients than in patients with either LC or HCC (*P* = 0.035 for CHB *vs.* LC; *P* = 0.020 for CHB *vs*. HCC). The data of further research showed that eRF3b/GSPT2 promoted the entrance of the HepG2 cells into the S-phase and that one of the substrates of the mTOR kinase, 4E-BP1, was hyperphosphorylated in eRF3b-overexpressing HepG2 cells.

**Conclusions:**

Overall, the differentially expressed protein eRF3b, which was discovered as a biomarker for HCC, could change the cell cycle and influence the phosphorylation status of 4E-BP1 on Ser65 in HepG2.

## Introduction

Worldwide, over 350 million individuals are chronically infected with hepatitis B virus (HBV) and 15–25% of them are at risk of developing and dying from HBV-related chronic liver disease, including cirrhosis and hepatocellular carcinoma (HCC) [1]. HBV infection and its sequelae are now recognized as serious problems globally. However, in clinical practice, one of the great inherent difficulties is deciphering how to screen hepatocellular carcinoma as early as possible. As we now know, about 90% of HCC cases develop on a background of liver cirrhosis (LC) [2]. It is therefore very important to diagnose the disease at an early stage, which should assist in better management of patient care.

The study of proteomic patterns (which is essentially an analysis of a panel of dozens to hundreds of mass spectrometry peaks), has been applied to screening disease states [3], [4], [5], [6]. It is well known that human serum is a complex medium that contains thousands of different types of proteins/peptides, many of which may be potential biomarkers for the clinical diagnosis of various fatal diseases. Recently, an affinity bead-based purification technique was developed that reduces cost and makes proteomic procedures suitable for general MS analysis [7].

In the present study, weak cation-exchange (WCX) magnetic bead purification and MALDI-TOF MS were used to assess protein expression profiles to search potential serum markers for LC or HCC. A 4210 Da protein, which expressed differently between the LC/HCC and CHB groups, was later identified as eukaryotic peptide chain release factor GTP-binding subunit 3b (eRF3b). The further analysis validated the differential expression of eRF3b in blood from healthy control and three stages of hepatitis B-related diseases. At the same time, we investigated the characteristics of eRF3b and found it can change the cell cycle and influnce the phosphoralation status of 4E-BP1.

## Results

### Biomarker discovery from HBV–related disease

Totally, 88 distinctive peaks were resolved. After compared the AHB and CHB samples, we found there were 49 peaks (proteins) differed significantly between acute and chronic patients. Each mean and standard deviation (SD) values were calculated. AUC was the area of ROC (receiver operator characteristic curve). Results for the top-ten markers were summarized in Table S1. Serum protein profiles were also compared among mild degree, moderate degree and severe degree hepatitis. We only selected the top-ten distinguished proteins according to their *P*-value. Results for the top-ten markers were summarized in Table S2.

Among the distinctive peaks, there are eight peaks (proteins) differed between the LC and CHB groups, 9 peaks (proteins) that differed significantly between the HCC and CHB groups. Only one peak (2093 Da) differed significantly between the HCC and LC groups. Among the distictive peaks for HCC, eight (88.8%) proteins had molecular weights (Mw) that were smaller than 5000 Da, and one (11.2%) protein had a Mw greater than 5000 Da. Because only one protein differed significantly between the HCC and LC groups, it was suggested that when patients converted to HCC from LC, the patient's serum proteomic profile was almost the same as with LC. This result indicated that it is likely more applicable in clinical use to search for potential LC markers from CHB patients than from HCC patients.

To assess the diagnostic efficacy, each mean and standard deviation (SD) value for the eight peaks was calculated. The cut-off value was defined as the mean plus one SD of the HCC group, which was then used to determine the respective sensitivities, specificities, and validation ability of the HCC markers. Results of the markers for HCC are summarized in Table 1.

**Table 1:** Determination of the sensitivity and specificity for the 9 HCC markers.

Mw(Da)	Intensity(mean±SD)/(arb.U) (HCC vs CHB)	Cutoff vaule	Sensitivity (%)	Specificity (%)	Validation (%)	AUC	*P* value
4268±2	77.67±36.91vs135.81±58.36	114.58	78.57	57.89	61.11	0.7979	5.43×10^−3^
4154±2	36.51±10.96vs52.11±21.62	47.47	85.71	57.89	62.22	0.7603	1.62×10^−2^
9288±2	1012.59±354.32vs1441.97±470.87	1366.91	92.86	60.53	65.56	0.7782	2.13×10^−2^
11243±2	22.56±6.32vs15.05±4.97	16.24	92.86	68.42	72.22	0.8318	2.13×10^−2^
4210±2	557.38±211.96vs807.11±339.65	769.34	71.43	67.11	67.78	0.7547	2.63×10^−2^
4195±2	95.14±35.09vs134.87±55.14	130.23	71.43	61.84	63.33	0.7641	2.63×10^−2^
4091±2	117.98±34.11vs157.38±53.46	152.09	71.43	53.95	56.67	0.7350	2.63×10^−2^
4169±2	52.96±19.51vs74.91±30.47	72.47	71.43	59.21	61.11	0.7413	2.63×10^−2^
2105±2	32.9±12.32vs45.74±18.02	45.22	85.71	50.00	55.56	0.7190	4.07×10^−2^

Notes: For each peak, the mean intensity and standard deviation (SD) were calculated. The cut-off value was defined as the mean plus one SD of those in the HCC group, which was then used to determine the sensitivities and specificities of the HCC. Sensitivity was defined as the ratio of HCC samples with a mass intensity less than cut-off value to all HCC samples, while the specificity was defined as the ratio of CHB samples with a mass intensity greater than the cut-off value to all CHB samples. Validation ability was defined as the ratio of samples with right diagnose according to cutoff value to all samples. AUC was the area of ROC (receiver operator characteristic curve).

### Identification of a 4210 Da protein

**Figure 1:**
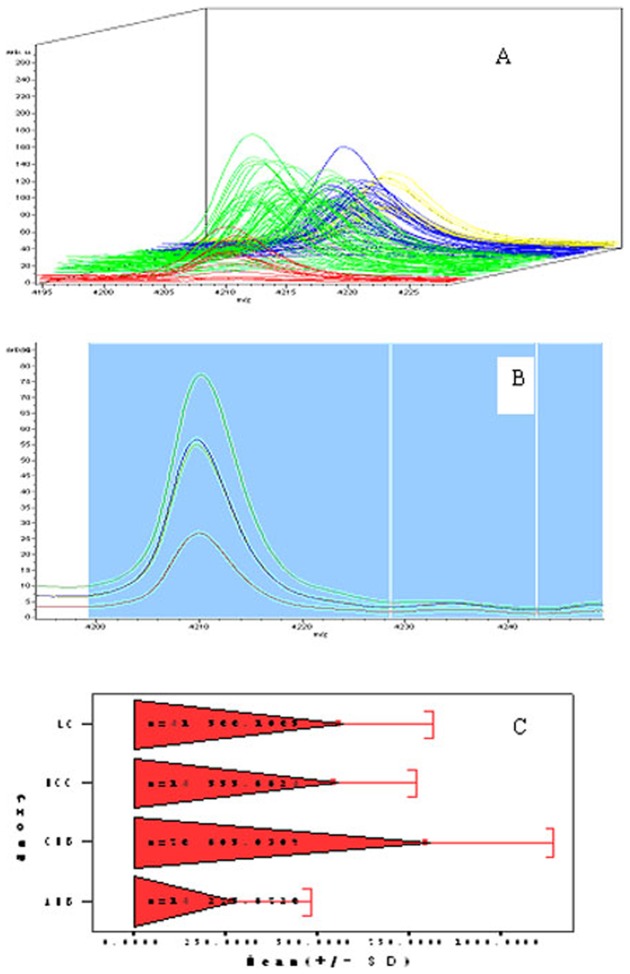
Serum protein profiles of a 4210 Da protein using MALDI-TOF mass spectrometry analysis. (A) Stack view of the 4210 Da protein in four groups analyzed by ClinProTools™ 2.1 software (red is AHB, green is CHB, blue is LC, yellow is HCC). X-axis is in m/z values, y-axis is in relative intensity units. (B) The average relative intensity of 4210 Da in four groups analyzed by ClinProtTools™ 2.1 software (red is AHB, green is CHB, blue is LC, yellow is HCC). The x-axis is in m/z values, y-axis is relative intensity units, and the bar-graph in (C) is drawn using SAS software.

**Figure 2:**
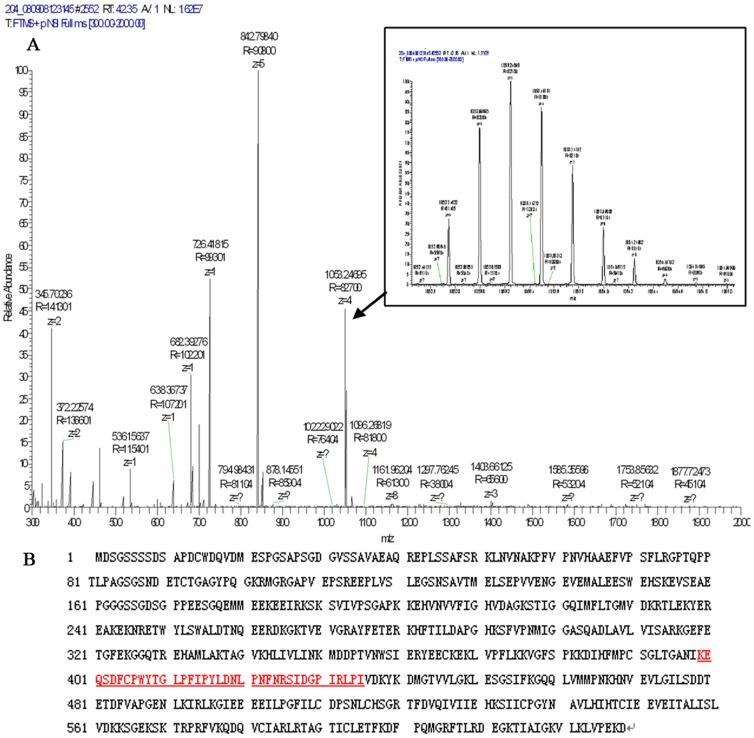
Identification of 4210 Da through MS/MS. (A) MALDI-TOF production spectrum of multi-charged peptides derived from m/z 4210. 1053.24(+4) was selected as the parent ion and its tandem MS/MS spectra are shown in the inset. (B) The peptide sequence that matched with eRF3b (Position:399–435) are underlined.

In this study, we discovered a 4210 Da protein that was different between the LC/HCC and CHB groups, but showed no significant difference between the HCC and LC groups (Fig. 1). Among the proteins bound to magnetic bead, small molecule are dominant. Although 11234 Da protein showed in Table 1 had higher sensitivity than 4210 Da protein, we payed close attention to low mass protein with molecular weight lower than 10 000 Da. In addition, the sensitivity, specificity, and the validation of 4210 Da protein were higher than other proteins with low molecule weight. Therefore, we next characterized the 4210 Da protein. After fractionation with WCX magnetic beads, the sample was subjected to LTQ Obitrap XL linear Ion Trap Mass Spectrometer (Fig. 2a; Thermo Fisher Scientific, Inc.). After the data were analyzed with Bioworks Browser 3.3.1 and searched using Sequest™, the 4210 Da protein was identified as eRF3b in the NCBI BLAST protein database (Fig. 2b).

### The tissue and blood expression of eRF3b/GSPT2

**Figure 3:**
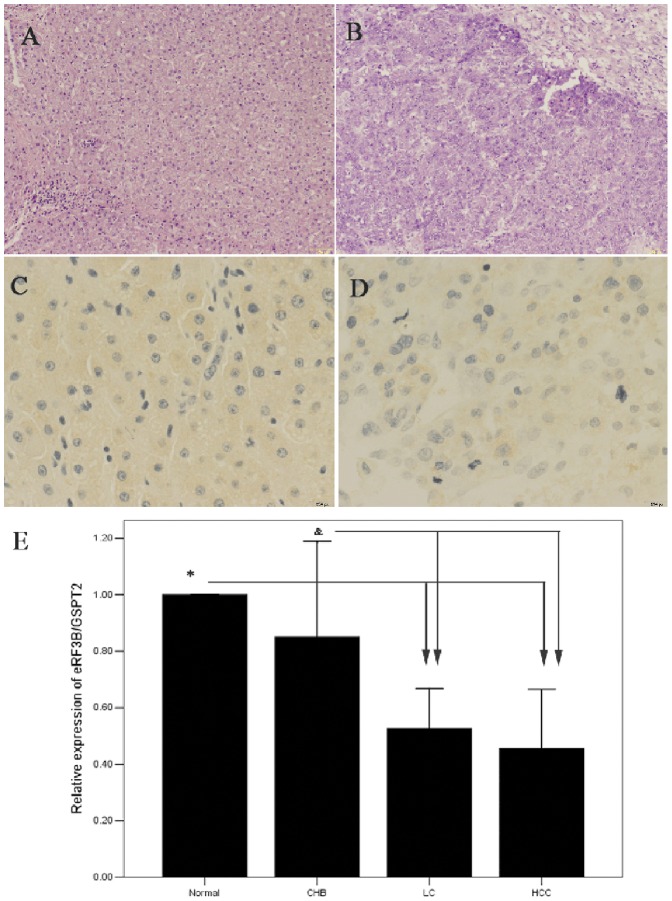
H&E staining, immunohistochemical analysis, and relative expression of GSPT2 mRNA in the blood of individuals with HBV-related diseases. (A) Normal liver tissue; (B) hepatocellular carcinoma tissue; (C) adjacent normal liver tissue; (D) hepatocellular carcinoma tissue; (E) relative expression of GSPT2 mRNA in the blood.

We first investigated the distribution of eRF3b in liver tissue. The results are shown in Fig. 3 (Fig. 3A and Fig. 3C for adjacent normal tissue; and Fig. 3B and Fig. 3D for carcinoma tissue), which indicated the positive expression of eRF3b in cytoplasm. To evaluate the relation between the level of eRF3b/GSPT2 expression and different developmental stages of disease, the HBV-related diseases were classified into groups as follows: CHB, LC and HCC. Relative over-expression of GSPT2 was detected in six of 12 samples from CHB patients, two from 22 LC, and one from 11 HCC when compared with 16 normal controls (Fig. 3E). In total, the mean expression of GSPT2/18s rRNA was 0.77, 0.54 and 0.48 in CHB, LC and HCC, respectively. The expression of the gene was higher in normal controls and CHB than in LC and HCC (*P* = 0.06 for normal *vs.* CHB; *P* = 0.035 for CHB *vs.* LC; *P* = 0.02 for CHB *vs.* HCC).

### The expression of eRF3b/GSPT2 in nine kinds of cells

**Figure 4:**
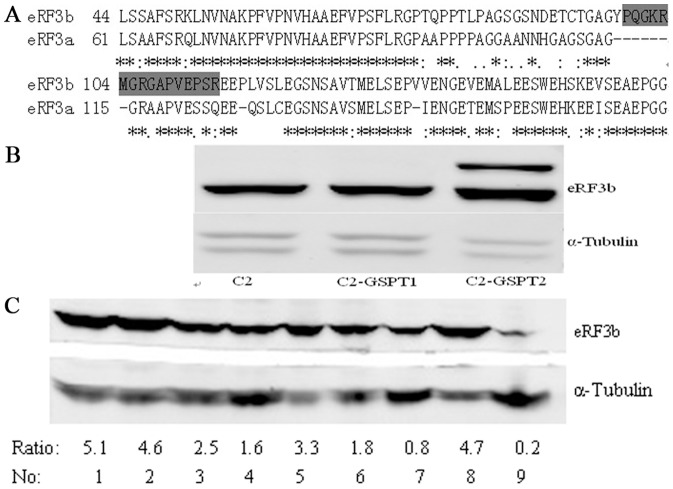
Expression of eRF3b protein in various cell lines. (A) Alignment of the predicted amino acid sequence of human eRF3a and eRF3b. The shaded peptide sequence was used for rabbit immunization. (B) Expression of eRF3b in cells transfected with pEFGP-C2 (C2), pEFGP-C2-GSPT1 (C2-GSPT1) and pEFGP-C2-GSPT2 (C2-GSPT2). (C) The ratios of eRF3b to α-tubulin expression in various kinds of cells (Lane 1 represents QSG-7701, followed by HepG2, HepG2.215, RD, Hela, Hek293T, RPE, MDA-MB-231, and U251, respectively).

Due to the high similarities in the C-terminal domains, the peptides for rabbit immunization were chosen in the divergent regions of the N-terminal domains (Fig. 4a). The synthetic peptides PQGKRMGRGAPVEPSR derived from the eRF3b protein sequence were used to immunize rabbits and we then retrieved the anti-eRF3b antibody. The specificities of the antibodies were tested by western blot analysis using extracts of human HepG2 cells overexpressing either eRF3a/GSPT1 or eRF3b/GSPT2 and of controlled cells. As shown in Fig. 4B, antibodies directed against eRF3b recognized a single band of 114 kDa in pEGFP-C2-*GSPT2* transfected cells and a bind of 87 kDa in pEGFP-C2*-GSPT1* transfected cell and pEGFP-C2 transfected cells.

The protein expression level of eRF3b/GSPT2 was analyzed in nine types of cell lines by western blotting. The ratios of eRF3b/GSPT2 to α-tubulin were 5.1, 4.6, 2.5, 1.6, 3.3, 1.8, 0.8, 4.7, 0.2, respectively (Fig. 4c) The results showed that eRF3b/GSPT2 was expressed differentially in cells, with the lowest level in the U251 cell line. Of the three types of hepatocyte cell lines, the protein expression level in the HepG2.215 cell line was the lowest, using α-tubulin as a control.

### Effect of eRF3b over-expression on the cell cycle and mTOR pathway

**Figure 5:**
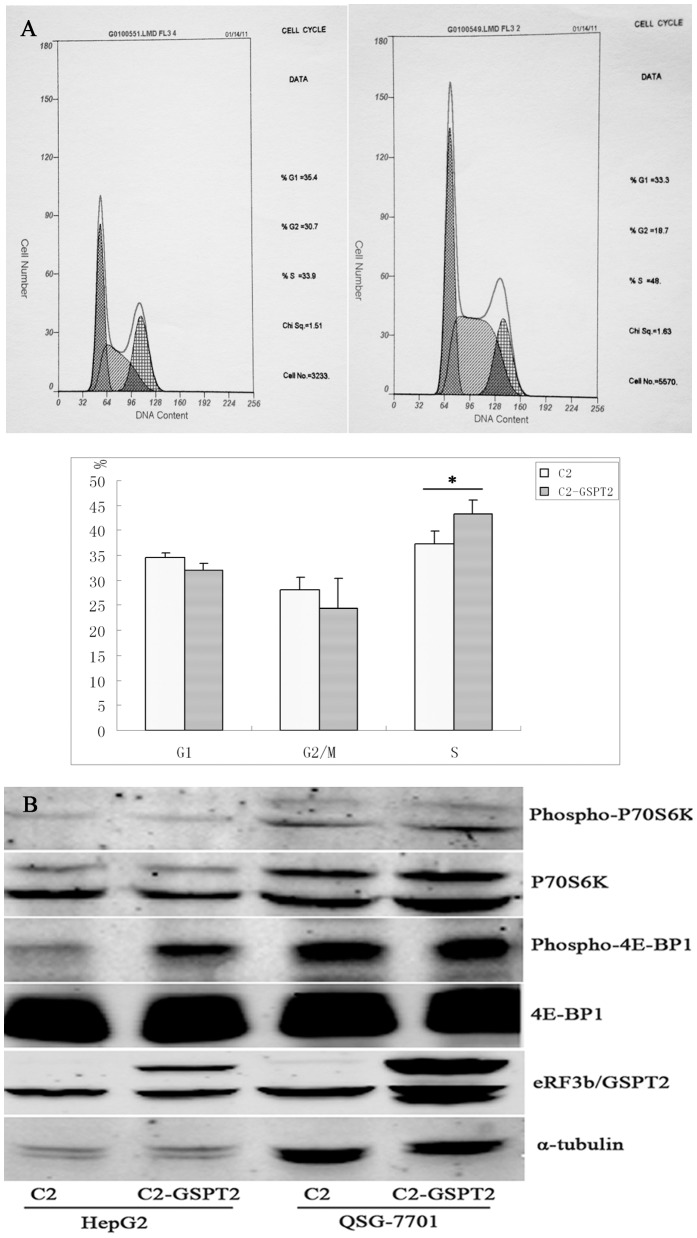
Effects of eRF3b overexpression on the cell cycle (A) and the phosphorylation statuses of mTOR targets (B).

Parallel cultures of HepG2 cells were transfected with either empty vector pEGFP-C2 or the pEGFP-C2-*GSPT2*, and the cell cycle was analyzed by flow cytometry. As shown in Fig. 5A, a clear decrease in the number of cells in G1 phase and G2/M and a concomitant increase in the number of cells in S phase were observed for cells transfected with pEGFP-C2-*GSPT2* compared to pEGFP-C2 control cells. The G1 phase percentage of pEGFP-C2 control cells and pEGFP-C2-*GSPT2* cells were 34.47±0.95 and 32.1±1.20, respectively (*P* = 0.055). The corresponding G2/M phase percentage were 28.20±2.50 and 24.5±3.80 (*P* = 0.242), respectively. S phase percentage were 37.37±2.45 and 43.40±2.60 (*P* = 0.043), respectively. It was suggested that eRF3b promoted the entrance of the HepG2 cells into the S-phase.

Phosphorylation events appear to play a leading role, via the mTOR signaling pathway, in the regulation of translational initiation and translation rates in a variety of cell systems [8]. p70S6K and 4E-BP1 are targets of mTOR and are positively regulated via phosphorylation [9]. Studies show that mTOR phosphorylation of p70S6K1 increases its kinase activity; and that phosphorylation of 4E-BP1 results in the dissociation of 4E-BP1 from eIF4E and the activation of cap-dependent translational initiation [8]. In order to study the effects of eRF3b overexpression on the mTOR pathway, the phosphorylation statuses of 4E-BP1 and p70S6K1 in pEGFP-C2- and pEFGP-C2-GSPT2-expressing cells were examined. Western blot was performed using antibodies directed against eRF3b, p70S6K1, phospho-p70S6K1 Thr389, 4E-BP1, phospho-4E-BP1 Ser65 or α-tubulin, which served as a loading control. The results showed a hyperphosphorylation of 4E-BP1 (Ser65) in eRF3b-overexpressing HepG2 cells compared to pEGFP-C2-expressing control cells, with no changes in QSG-7701 cells (Fig. 5B), which indicated that the effect of eRF3b/GSPT2 on phosphorylation status of 4E-BP1 took place in hepatoma carcinoma cells but not in normal cells. However, more cell lines need to be tested to validate the results.

## Discussion

Proteomic analyses based on mass spectrometric techniques are still innovative ways to identify the components of protein complexes in serum, and have been successfully employed in the discovery of new biomarkers in different human diseases [10], [11]. In this study, we directly profiled protein/peptide patterns and determined several potential markers that discriminated HCC from CHB samples. Since more than 90% of HCC cases have a background of cirrhosis, it is very important to be able to distinguish LC from CHB cases. Moreover, only one peak (2093 Da) that differed significantly was found when comparing HCC and LC groups, which implied that there was almost no difference in the serum proteomes between the HCC and LC groups. In other words, in clinical use it is more efficient and practical to screen HCC patients from a CHB group.

In this study, the MS results identified two differentially expressed proteins, the 4210 Da protein and the 1860 Da protein. Although their roles in the pathologic mechanisms of action in HBV-related diseases are still unknown, they can be used as potential biomarkers in diagnosis and prognosis. The 1860 Da protein was reported previously [12]. The 4210 Da protein was later identified as “GSPT2 eukaryotic peptide chain release factor GTP-binding subunit ERF3”. We know that the termination of protein synthesis in eukaryotes involves at least two polypeptide release factors (eRFs), eRF1 and eRF3. In mammals, the two genes encoding eRF3 (eRF3a and eRF3b) are structural homologues and have been identified and named GSPT1 and GSPT2. ERF3a and eRF3b are now known to differ in their N-terminal domains. Chauvin [13] indicated that eRF3a is the major factor that acts in translational termination in mammals, and that eRF3b can substitute for eRF3a in this function. In order to quantify the mRNA expression level, we collected blood samples, extracted the RNA and then detected the expression of GSPT2 using real-time PCR. It was found that the GSPT2 was differentially expressed in three types of HBV-related disease. Its expression was higher in the blood of normal controls and CHB patients compared with LC and HCC patients, which was in agreement with the MS results. Our results showing that GSPT2 was expressed in blood is to an extent consistent with the results of Hoshino et al. [14].

Eukaryotic release factors are encoded by two distinct genes [14], [15], [16], eRF3a/GSPT1 and eRF3b/GSPT2, which are located on human chromosome 16 and X, respectively. They share 87% identity for both mRNA and protein sequences. eRF3 has been reported to be involved in translational termination [17], [18], cell-cycle regulation [19], [20] and other cellular processes such as cytoskeleton organization and tumorigenesis [21]. A recent report showed that eRF3a depletion induced G1 phase arrest, and that the translation rate decreased via inhibition of mammalian TOR (mTOR) activity, indicating that eRF3a regulates mTOR activity [22]. The protein kinase TOR is known to be a major effector of cell growth through its regulation of protein synthesis [8], [23]. mTOR controls protein synthesis through phosphorylation and inactivation of initiation factor 4E-binding protein 1 (4E-BP1), and through the phosphorylation and activation of ribosomal protein S6 kinase 1 (S6K1). In this study, we investigated the phosphorylation statuses of two kinases of 4E-BP1 and p70S6K1 in the mTOR pathway. We found that the up-regulation of eRF3b phosphorylates 4E-BP1 on Ser65 in the HepG2 cell line, suggesting that eRF3b/GSPT2 contributes to translational initiation and cellular growth by phosphorylation of 4E-BP1. However, the effect of eRF3b on 4E-BP1 was not observed in the QSG-7701 cell line. Although eRF3b and eRF3a show 87% mRNA and protein sequence identity, the function of eRF3b is still unclear. It has been shown that 4E-BP1 phosphorylation and stability can be regulated by multiple kinases from various cell-signaling pathways [24]; however, whether the effect of eRF3b on 4E-BP1 is regulated by the mTOR signaling pathway or other pathways, requires further investigation.

In conclusion, we suggest that eRF3b/GSPT2, which differentially expressed in CHB and LC/HCC and influence the phosphorylation status of 4E-BP1, might be an important biomarker for HCC.

## Materials and Methods

### Chemicals and antibodies

All chemicals and solvents used were purchased from Sigma–Aldrich (St. Louis, MO). Solvents were HPLC grade and were used without further purification. Alpha-cyano-4-hydroxycinnamic acid (CHCA) was used as the MALDI matrix. WCX magnetic beads and Standard Preparation (Peptide Calibration Standard #206195 and Protein Calibration Standard I #206355) were purchased from Bruker Daltonik GmbH (Bremen, Germany).

The anti-α-tubulin antibody was from Epitomic Inc (Burlingame, California). Antibodies directed against 4E-BP1, phospho-4E-BP1 (Ser65), p70S6K1 and phospho-p70S6K1 (Thr389) were from Cell Signaling Technology, Inc (Danvers, USA). An IRDye 680-conjugated goat anti-rabbit IgG secondary antibody was from Rockland Immunochemical Inc (Gilbertsville, PA). The anti-eRF3b antibody directed against the human eRF3b was produced by Sangon (Shanghai, China).

### Serum samples and blood samples

Serum samples from patients with hepatitis B were collected at the Fifth Hospital in Shijiazhuang City, Hebei Province, China. Serum from 74 chronic hepatitis B (CHB) patients (mean age, 39.14±14.10 years; range, 12-76 years), 41 LC patients (mean age, 37.25±13.62 years; range, 29-58 years) and 14 HCC patients (mean age, 46.53±11.14 years; range, 22-63 years) were collected for MALDI-TOF-MS. In addition, blood samples from 16 normal controls, 12 CHB, 28 LC and 16 HCC were collected for qRT-PCR.

All patients were diagnosed according to the Manual of CHB Prevention and Cure (2005, 12. China). Some of the CHB and LC patients were confirmed by pathology, and all HCC patients were confirmed by pathology. Exclusion standard: complicating hepatitis A, C, E, alcoholic liver disease, autoimmune liver disease and drug liver disease. All patients gave written informed consent and the study was approved by the ethics committee of Hebei Medical University.

### Magnetic bead-based method for MALDI-TOF MS

The contents of the binding, washing, and elution solutions provided by Bruker Daltonik GmbH as part of the kits are proprietary. Briefly, 10 µL of a MB–WCX binding solution and 5 µL serum were transferred to a 0.2-ml thin-walled PCR-tube (ABgene, UK). A 10-μL homogenous magnetic particle solution was added, and mixed and left for 5 min. The tubes were placed in a 2×8 well magnetic bead separator (MBS) for 30 s for magnetic fixation of the MB–WCX particles. The supernatant was aspirated and the tubes were removed from the MBS device. Wash solution (100 µL) was added and carefully mixed with the magnetic beads. The tube was then replaced in the MBS device and moved back and forth sequentially between adjacent wells on each side of the magnetic bar in the MBS device. This facilitated washing of the magnetic particles as they are fixed to the tube wall, moving them through the washing solution in succession. After fixation of the magnetic beads for 30 s in the MBS device, the supernatant was aspirated. This washing procedure was repeated three times. After the final washing step, bound molecules were eluted by incubation with 5 µL of MB–WCX elution solution for 1 min before collecting the eluate using the MBS device. Finally, 5 µL MB–WCX stabilization solution was added to the eluate.

The eluate (1 µL) was then mixed with 10 µL of matrix solution (0.3 g/l HCCA in ethanol: acetone 2∶1), and 1 µL was spotted onto a 600-μm diameter spot size 384 AnchorChip™ target plate (Bruker Daltonik GmbH) and left to dry. For each run a new HCCA-matrix was prepared. A protein calibration standard (Protein Calibration standard 1, Bruker Daltonik GmbH) was dissolved in 125 µL 0.1% aqueous TFA, and 0.5 µL of the solution was applied to target spots in close proximity to the serum samples for external calibration. MALDI-TOF MS experiments were performed using an AutoflexIII (Bruker Daltonik GmbH) in a LP-Clinprot model. Before each acquisition cycle the position was pretreated with 10 laser shots at 74% laser power to improve spectra quality. Determine the peak m/z values or intensities in the mass range of 1000–12 000 Da, focusing on mass range 1000–10 000 Da. For systemic errors, a ±2 Da mass accuracy for each spectrum was tolerated [25].

### Immunohistochemical analysis

The sections of liver tissues were deparaffinized by incubation at 60°C for 24 h, followed by two successive immersions in xylene at 56°C for 30 min each, followed by hydration in solutions with decreasing concentrations of ethanol (100%, 95%, 80%, and 70%). For antigen retrieval, the slides were incubated in 10 mM citrate buffer (pH 6.0) in a pressure cooker for 30 min, after preheating for 14 min. Endogenous peroxidase activity was blocked by incubating with a 3% peroxide/methanol solution for 15 min at room temperature. Other steps were performed according to the protocol of a commercially available Histostain–plus kit (ZSGB-BIO, Beijing, China).

### RNA extraction and real-time PCR

Blood RNA was extracted using the Total RNA Isolation System (Bioteke, Beijing, China) and the first-strand cDNA was synthesized using the ReverAid™ First Stand cDNA synthesis Kit. Expression of the eRF3b/GSPT2 was investigated using real-time quantitative RT-PCR based on SybrGreen fluorescence methodology. The forward primer (F) for GSPT2 gene was “ttgctgccttaaccccgccg” and the reverse primer (R) was “tgctgctgctgcccaatcc”. The forward primer for 18s rRNA was “cagccacccgagattgagca” and the reverse primer was “tagtagcgacgggcggtgtg”. Human 18S rRNA was used as the endogenous housekeeping control gene. All reactions were performed in triplicate and included a negative control, in which template was not added. PCR reactions were performed in a Corbett Rotor gene 6000 System (Sydney, Australia). Cycling conditions were as follows: 5 minutes at 95°C; and 40 cycles of 15 seconds at 95°C, 15 seconds at 59°C and 15 seconds at 72°C. Relative quantification of the mRNA levels for GSPT2 were determined using the CT method [26].

### Plasmid construction, cell culture and transient transfection

For plasmids pEGFP-C2-*GSPT2* and pEGFP-C2-*GSPT1*, the genes GSPT2 and GSPT1 were cloned and digested with BamHI and ECORI (NEB) ends, and then inserted into the pEGFP-C2 vector (AMICON) linearized by BamHI and ECORI. The human liver hepatocellular carcinoma cell line HepG2 and normal human liver cell line QSG-7701 were cultured in Dulbecco's modified Eagle's medium (DMEM, Hyclone Laboratory, Logan, UT) supplemented with 10% fetal calf serum and 1% penicillin-streptomycin at 37°C in a 5% CO_2_ atmosphere. The cells were transfected with purified recombinant plasmids using Turbofect™ in vitro transfection reagent (Fermentas, Canada) according to the manufacturer's instructions.

### Flow cytometry analysis

Cells were plated in 60-mm dishes and were transfected with pEGFP-C2-*GSPT2* and pEGFP-C2 was used as control. Twenty-four hours later, cells were collected and fixed in 0.5% paraformaldehyde for one hour and then in 70% ethanol. After washing with PBS, cells were resuspended in PBS containing 1 mg/ml RNase and 50 g/ml propidium iodide, incubated for 20 min in the dark at room temperature, and then analyzed by flow cytometry using a Coulter Epics (Beckman, Fullerton CA).

### Data collection and analysis

The MS data were collected with FlexControl^MS^ 3.0 software and analyzed with Flex Analysis 3.0 software, ClinProTools™ 2.1 software (Bruker Daltonik GmbH), include detecting peak intensities of interest and compiling the peaks across the spectra obtained from all samples. Calibration was performed using Standard Preparation. Distinguished proteins were identified by a LTQ Obi trap XL linear Ion Trap Mass Spectrometer, which was produced by Thermo Fisher Scientific, Inc. (Waltham, MA). The profiles were analyzed using Biowork Browser 3.3.1 and searched with Sequest™. Statistical analysis was performed using one-way ANOVA followed by SNK test for multiple-comparisons or Kruskal-Wallis test for nonparametric test using SAS 9.1.3 (SAS Institute Inc., site number 56955002). Statistical significance was set at *P*<0.05.

## Supporting Information

Table S1 **Determination of the sensitivity and specificity for the top-ten AHB markers.**
(DOC)

Table S2 **The top ten distinguished proteins between different degrees of chronic hepatitis B.**
(DOC)
