# Supplementary material for: eRF3b, a Biomarker for Hepatocellular Carcinoma, Influences Cell Cycle and Phosphoralation Status of 4E-BP1
Source: PLoS One. 2014 Jan 23;9(1):e86371. doi: 10.1371/journal.pone.0086371 (PMC3900531; doi:10.1371/journal.pone.0086371)
Supplement: Table S1 — Determination of the sensitivity and specificity for the top-ten AHB markers. (DOC) [file pone.0086371.s001.doc]

Table S1 Determination of the sensitivity and specificity for the top-ten AHB markers

| Mw (Da) | Intensity(mean±SD)/(arb.U) (AHB vs CHB) | Cutoff vaule | Sensitivity (%) | Specificity (%) | Validation (%) | AUC | *P* value |
| --- | --- | --- | --- | --- | --- | --- | --- |
| 4154±2  4268±2  4091±2  3952±2  4123±2  2105±2  3192±2  4210±2  4169±2  2769±2 | 22.08±9.15vs52.23±21.73  49.54±27.95vs135.77±58.55  70.29±26.13vs150.19±51.1  53.1±14.28vs106.68±54.27  33.43±10.72vs64.21±24.55  19.24±10.15vs47.99±18.57  42.79±21.68vs102.36±36.66  276.81±204.06vs807.04±339.44  26.79±17.19vs70.45±28.63  15.2±8.11vs35.83±18.73 | 40.38  105.44  122.55  81.66  54.87  39.54  86.15  684.93  61.17  31.42 | 7.1  100  100  100  100  100  100  100  100  100 | 100.00  68.42  68.42  64.47  63.16  64.17  64.17  72.37  67.11  51.32 | 85.56  73.33  73.33  70.0  68.89  70.00  70.00  76.67  72.22  58.89 | 0.9144  0.9342  0.9286  0.8863  0.8703  0.9380  0.9192  0.9088  0.9079  0.8665 | ＜10^-6^  ＜10^-6^  ＜10^-6^  ＜10^-6^  ＜10^-6^  ＜10^-6^  ＜10^-6^  ＜10^-6^  ＜10^-6^  ＜10^-6^ |
