# Supplementary material for: eRF3b, a Biomarker for Hepatocellular Carcinoma, Influences Cell Cycle and Phosphoralation Status of 4E-BP1
Source: PLoS One. 2014 Jan 23;9(1):e86371. doi: 10.1371/journal.pone.0086371 (PMC3900531; doi:10.1371/journal.pone.0086371)
Supplement: Table S2 — The top ten distinguished proteins between different degrees of chronic hepatitis B. (DOC) [file pone.0086371.s002.doc]

Table S2 The top ten distinguished proteins between different degrees of chronic hepatitis B

| Mass（Da） | Intensity (mean±SD)/(arb.U) | AUC | P |
| --- | --- | --- | --- |
|  | Mild degree vs Moderate degree vs Severe degree |  |  |
| 1779±2 | 68.91±37.25 vs 98.16±42.55 vs 26.06±7.71 | -- | ＜1.0E-6 |
| 1691±2 | 35.6±19.73 vs 49.58±23.98 vs 13.71±4.2 | -- | ＜1.0E-6 |
| 1450±2 | 53.03±30.94 vs 58.79±29.91 vs 21.28±8.15 | -- | ＜1.0E-6 |
| 1564±2 | 17.81±7.21 vs 20.77±6.32 vs 11.42±2.81 | -- | 1.0E-6 |
| 1866±2 | 109.34±82.84 vs 165.6±122.49 vs 23.98±7.65 | -- | 1.2E-5 |
| 1349±2 | 18.84±8.14 vs 21.02±7.47 vs 10.96±4.18 | -- | 2.2E-5 |
| 1212±2 | 7.37±2.83 vs 7.97±3.65 vs 4.19±1.6 | -- | 5.0E-5 |
| 5754±2 | 40.05±17.59 vs 31.71±13.92 vs 59.95±25.53 | -- | 6.4E-4 |
| 2545±2 | 22.49±8.61 vs 18.45±6.92 vs 30.68±11.38 | -- | 1.6E-3 |
| 4363±2 | 17.54±10.06 vs 18.19±7.5 vs 29.28±13.33 | -- | 8.1E-3 |
|  | Mild degree vs Severe degree |  |  |
| 1779±2 | 68.9±37.24 vs 26.06±7.7 | 0.9280 | 2.1E-6 |
| 1691±2 | 34.61±19.63 vs 12.77±4.09 | 0.9156 | 3.4E-6 |
| 1212±2 | 6.87±2.69 vs 3.85±1.47 | 0.8449 | 2.2E-4 |
| 1866±2 | 111.37±83.99 vs 25.12±7.82 | 0.9318 | 6.1E-4 |
| 1450±2 | 51.3±31.03 vs 21.62±8.21 | 0.8697 | 6.1E-4 |
| 1349±2 | 18.77±8.11 vs 10.88±4.15 | 0.8251 | 1.6E-3 |
| 1564±2 | 18.83±7.31 vs 12.38±2.98 | 0.8238 | 3.6E-3 |
| 5805±2 | 144.5±89.93 vs 279.82±191.86 | 0.8300 | 1.4E-2 |
| 2687±2 | 23.92±8.05 vs 35.87±16.53 | 0.7754 | 1.4E-2 |
| 4363±2 | 21.56±10.99 vs 33.44±14.73 | 0.7419 | 1.4E-2 |
|  | Moderate degree vs Severe degree |  |  |
| 1779±2 | 95.81±42.64 vs 24.39±7.09 | 0.9983 | 1.1E-4 |
| 1691±2 | 49.96±24.01 vs 14.03±4.28 | 0.9898 | 1.7E-4 |
| 5755±2 | 24.03±10.41 vs 44.31±16.94 | 0.8795 | 1.7E-4 |
| 1564±2 | 21.1±6.57 vs 11.75±2.82 | 0.9423 | 2.1E-4 |
| 1349±2 | 21.1±7.48 vs 11.04±4.17 | 0.9100 | 3.8E-4 |
| 2545±2 | 18.44±6.97 vs 30.71±11.45 | 0.8455 | 4.9E-4 |
| 1450±2 | 57.51±29.39 vs 20.31±7.97 | 0.9287 | 5.7E-4 |
| 1866±2 | 164.4±121.9 vs 23.25±7.51 | 0.9847 | 1.4E-3 |
| 2687±2 | 21.01±10.68 vs 36.24±16.83 | 0.8183 | 1.9E-3 |
| 1212±2 | 8.28±3.79 vs 4.37±1.72 | 0.8574 | 4.5E-4 |

Note：There was no difference between Mild degree and Moderate degree of CHB patient’s serum protein profile.
